# The impact of FDA and EMEA guidelines on drug development in relation to Phase 0 trials

**DOI:** 10.1038/sj.bjc.6603925

**Published:** 2007-08-28

**Authors:** S Marchetti, J H M Schellens

**Affiliations:** 1 Division of Clinical Pharmacology, Department of Medical Oncology, The Netherlands Cancer Institute, Plesmanlaan 121, 1066 CX Amsterdam, The Netherlands

**Keywords:** phase 0 trial, drug development, microdose, pharmacokinetics, pharmacodynamics, toxicity

## Abstract

An increase in the number of identified therapeutic cancer targets achieved through recent biomedical research has resulted in the generation of a large number of molecules that need to be tested further. Current development of (anticancer) drugs is a rather inefficient process that for an average new molecule takes around 10–15 years. It is also a challenging process as it is associated with high costs and a low rate of approval. It is known that less than 10% of new molecular entities entering clinical Phase I testing progress beyond the investigational programme and reach the market; this probability is even lower for anticancer agents. In 2003, the US Food and Drug Administration (US FDA) declared the urgent need for new toolkits to improve the critical development path that leads from scientific discovery to the patient. In this scenario, Phase 0 (zero) trials should allow an early evaluation in humans of pharmacokinetic and pharmacodynamic profiles of test compounds through administration of sub-pharmacological doses and for a short time period to a low number of humans. Typically, Phase 0 studies have no therapeutic or diagnostic intent. Owing to the low doses administered and the low risk of toxicity, shorter preclinical packages to support these studies are required. Phase 0 trials have been proposed to help in making an early selection of promising candidates for further evaluation in Phase I–III trials, providing a potentially useful instrument for drug discovery, particularly in the field of oncology. Phase 0 studies are expected to reduce costs of drug development, and to limit the preclinical *in vitro* and *in vivo* testing and the time period of drug development. However, there are also concerns about the utility and feasibility of Phase 0 studies. In January 2006, guidelines on exploratory investigational new drug studies in humans have been published by the US FDA, and currently a Phase 0 programme is ongoing at the National Cancer Institute to evaluate the impact (feasibility and utility) of Phase 0 studies on drug development. In Europe, a Position Paper produced by the Evaluation of Medicinal Products (EMEA) in 2004 raised the possibility of a reduced preclinical safety package to support early microdose clinical studies, and, as announced by a recent Concept Paper on medicinal products published by the committee for medicinal products for human use of the EMEA, EMEA's guidelines on Phase 0 studies are expected shortly. The true impact of Phase 0 studies on the drug development process as well as on the safety needs to be carefully explored.

Drug development is a long, complex and expensive process. The conventional sequence of processes involved in drug development comprises a preclinical phase and three clinical phases, Phases 1, 2 and 3. Currently, the typical development for investigational new drugs takes between 10 and 15 years and is associated with high costs and low rate of approval (DiMasi *et al*, 2003). Owing to the fast progress in biomedical and pharmaceutical research, the number of new molecular entities starting the development process has increased significantly since about 1990; however, the rate of approval for marketing is declining: in 2005, only 20 new drugs were approved by the US Food and Drug Administration (US FDA), compared with 36 in 2004 and 53 in 1996 (Twombly, 2006). It has been estimated that currently a novel compound entering clinical Phase I testing has only an 8% chance of reaching the market, and the probability is even lower for an anticancer drug (Food and Drug Administration, 2006, http://www.fda.gov/oc/initiatives/criticalpath/whitepaper.pdf; Kola and Landis, 2004). This low success rate has been ascribed to the high number of new molecular entities with diverse mechanisms of action (MOA) to be tested for activity and efficacy combined with the lack of predictive preclinical models and inadequate and complex clinical trial designs. Overall 75% of the costs of drug development is associated with failures mainly in the early stages of development (Goodall, 2004). Often the toxicity is severe and poorly predictable. Furthermore, 40% of exits from Phase I trials are caused by inappropriate pharmacokinetics of the test compound (DiMasi, 2001, 2002); Wang and Urban, 2004). Several investigational new drugs fail in clinical testing because they do not behave pharmacologically as predicted in animal studies. Consequently, there is a need for additional methods to evaluate potential new drugs and to optimise existing development strategies. This is particularly true for the field of oncology where the increased understanding of genetic and molecular mechanisms involved in malignant cellular transformation has led to major changes in therapeutic approaches. Anticancer drugs able to inhibit specific targets responsible for malignant cellular transformation (e.g. genes, proteins, growth factors and receptors) and involved in specific pathways (e.g. angiogenesis, apoptosis, signal transduction, metastasis, drug resistance, etc.) have been discovered. Moreover, the application of combinatorial chemistry further increased the number of potential therapeutic agents (Leonard *et al*, 2001). In contrast with the traditional nonspecific cytotoxic antiproliferative agents, which often have a small therapeutic window, steep dose–toxicity curve and an efficacy assumed to be somehow related to toxicity, molecularly targeted agents usually show less toxicity, a wider therapeutic window and an efficacy more related to growth inhibition than to tumour shrinkage (Fox *et al*, 2002; Workman *et al*, 2006). Clearly, strategies used for the development of cytotoxic agents may not be suitable for molecularly targeted agents.

Consequently, new tools and strategies to make the drug development process faster and more efficient are being explored. In view of this, Phase 0 clinical studies have been proposed to modernise and optimise the clinical trial process (Kummar *et al*, 2007).

## PHASE 0 CLINICAL TRIALS

Phase 0 trials are clinical studies conducted early in Phase I, before the traditional dose escalation, safety and tolerance studies. These first-in-man trials should involve a very limited number of normal volunteers or patients, exposed to a novel compound at a reduced dose compared to starting doses in Phase I and for a short time period. Phase 0 clinical trials have no therapeutic or diagnostic purpose for the volunteer; in principle, they should allow researchers to quickly establish whether a novel compound has appropriate pharmacokinetic and pharmacodynamic profiles in humans. Phase 0 trials will not replace the traditional dose escalation, safety and tolerance studies and they will not indicate whether a candidate drug has a positive impact on the targeted disease. However, owing to the low doses administered, the limited number of humans treated and the reduced risk of toxicity, the Phase 0 strategy would require fewer preclinical *in vitro* and *in vivo* studies than a typical Phase I trial and a reduced amount of the experimental drug. Potentially, Phase 0 clinical studies could help in eliminating candidate drugs before they reach Phase I testing, thus reducing costs and time and improving the efficiency of drug development.

## EVALUATION OF MEDICINAL PRODUCTS GUIDANCE

In 2003, the European Agency for the Evaluation of Medicinal Products (EMEA) published a concept note followed by a Position Paper on the nonclinical safety studies needed to support human clinical trials with a single dose of a pharmacologically active compound using microdose techniques (CPMP/SWP/2599/02/Rev1, http://www.emea.europa.eu/pdfs/human/swp/259902en.pdf).

The Paper raises the possibility of a reduced preclinical safety package for sub-pharmacological (micro)dose clinical studies. The proposals of the EMEA are a major step forward in the recognition of the potential utility of microdose Phase 0 trials in improving the drug development process. As intended by EMEA, the aim of microdose studies is to evaluate human plasma pharmacokinetics and/or distribution properties or receptor selectivity profile of new drug candidates as early as possible in the preclinical stage of development. Theoretically, microdose trials should allow an early selection between promising and inappropriate molecules for further development.

In microdose trials, sub-pharmacological single doses (in the low microgram range) of test drug are administered to a small number of humans (healthy volunteers or patients). Ultrasensitive analytical methods (e.g. positron emission tomography (PET), accelerated mass spectrometry (AMS)) are needed to assess human exposure and drug distribution to estimate the pharmacokinetics of higher, clinically relevant doses, assuming linear pharmacokinetics.

The Paper defines a microdose as less than 1/100th of the dose of the test substance calculated from *in vitro* and animal models to yield a pharmacological effect, with a maximum dose of ⩽100 *μ*g. Microdose clinical trials may be conducted with a single test substance or with several closely related molecules to choose the more promising candidate or formulation.

As indicated in the Position Paper, an extended single-dose toxicity study in only one appropriate mammalian species with a control group is considered sufficient to support a microdose human trial if justified by comparative *in vitro* data. A sufficient number of animals to guarantee reliable interpretation of the results should be treated and two routes of administration (the intravenous and the clinically intended, when different from intravenous) are recommended. Animals should be observed for 14 days with an interim necropsy on day 2. Haematologic, clinical chemistry and histopathologic data as well as information regarding other relevant organ systems should be collected after 2 and 14 days at minimum. Gross necropsy of all animals is recommended. Notably, as indicated in the Position Paper, a safety factor of 1000 should be used to calculate the limit dose from animals to humans. *In vitro* genotoxicity tests are required, although under certain circumstances they can be limited. Moreover, all preclinical safety studies should be performed in accordance with the principles of Good Laboratory Practice.

EMEA's guidelines on Phase 0 trials are currently being prepared. In March 2006, the committee for medicinal products for human use (CHMP) of the EMEA published a Concept Paper about the development of a CHMP guideline on the nonclinical requirements to support early Phase I clinical trials with new pharmaceutical compounds (CHMP/SWP/91850/2006, http://www.emea.europa.eu/pdfs/human/swp/9185006en.pdf). A revised toxicology package designed to support Phase 0 trials without compromising human safety has already been discussed by the CHMP Safety Working Party (SWP) and the preparation of the guideline is ongoing. As anticipated in the Concept Paper, the guidance is intended ‘to facilitate a directed exploration of the physiology, pharmacology and/or pharmacokinetics of one or more candidate pharmaceutical products in humans’ in order ‘to optimise the selection of safer, more effective therapeutics for further development and ultimately make them available to patients sooner’. The guidance will describe early Phase I exploratory (Phase 0) approaches. These studies should evaluate pharmacokinetic and/or pharmacodynamic properties of the test molecule, possibly using a sensitive biomarker of activity. A dose–exposure relationship in humans could be obtained too. The guidelines will standardise and facilitate the requirements for Phase 0 trials and should allow flexibility of approaches.

Phase 0 clinical studies were not mentioned in the revised guidelines on the evaluation of anticancer medicinal products in humans published by the CHMP in December 2005 (CPMP/EWP/205/95/Rev.3/Corr.2, http://www.emea.eu.int/pdfs/human/ewp/020595en.pdf), although it has been proposed that one of the most interesting fields of application of Phase 0 trials could be the discovery/development process of new molecularly targeted anticancer compounds. However, current EMEA's guidelines on the clinical investigation of anticancer agents provide different opportunities for the evaluation of cytotoxic and non-cytotoxic anticancer compounds. These guidelines allow large flexibility in designing Phase I/II exploratory trials, provide opportunities to perform early trials in healthy volunteers besides cancer patients, introduce new end points (such as determination of a biologically effective dose) and encourage the evaluation of pharmacodynamic parameters previously not explored (e.g. biochemical and immunological parameters, functional imaging, proteomics, etc.). Therefore, we believe that most (if not all) issues explored by Phase 0 trials could already be addressed based on the outlined EMEA guidance and by an adequate Phase I study strategy, but official guidelines on Phase 0 studies could standardise and facilitate this new strategy in the evaluation of novel anticancer drugs.

## US FOOD AND DRUG ADMINISTRATION GUIDANCE

In the Critical Path Report published on March 2004, the US FDA denounced the ‘slowdown, instead of the expected acceleration, in innovative medical therapies reaching patients’ owing to a medical product development path ‘increasingly challenging, inefficient and costly’. US Food and Drug Administration declared the urgent need for new toolkits to modernise the critical development path that leads from scientific discovery to the patients. The aim was to improve the medical product development process to speed up ‘robust development pathways that are efficient and predictable and result in products that are safe, effective and sooner available to patients’ (Food and Drug Administration, 2006, http://www.fda.gov/oc/initiatives/criticalpath/whitepaper.pdf).

Following these concepts in April 2004, US FDA published a draft guidance regulating early human screening studies, and in January 2006 new industry guidelines for early exploratory drug studies in humans have been issued (http://www.fda.gov/cder/guidance/7086fnl.htm). The aim of the guidance, developed by the Center for Drug Evaluation and Research (CDER) (2006), was to ‘clarify what preclinical and clinical approaches, as well as chemistry, manufacturing and controls information, should be considered when planning exploratory studies in humans, including studies of closely related drugs or therapeutic biological products, under an investigational new drug application’. The guidelines defined also the characteristics and the aim of investigational new drug studies. In brief, they are defined as clinical trials conducted early in Phase I of clinical development in a very limited number of patients or healthy volunteers. The duration of dosing of the candidate compound should be limited (e.g. 7 days). These studies should not have any therapeutic or diagnostic purpose. The aim of Phase 0 studies is to explore pharmacokinetic and pharmacodynamic profiles of novel compounds in humans, to confirm in humans a mechanism of action (MOA) previously observed in experimental systems and to help in the early selection of the most promising lead product from a group of candidates interacting with a particular therapeutic target.

Interestingly, US FDA's guidance does not issue new regulations, but is an interpretation of existing recommendations on drug development. In particular, knowing that limited early Phase I studies were often supported by a more extensive preclinical database than was required by the regulations, the guidance addresses not only chemistry and manufacturing, but also the level and the type of preclinical safety testing recommended for an exploratory investigational new drug application.

The guidance describes in detail also three types of Phase 0 studies: clinical studies of pharmacokinetics or imaging (also noted as microdose studies), clinical trials to study pharmacologically relevant doses and clinical studies of MOAs related to efficacy.

*Microdose or imaging studies* are designed to evaluate pharmacokinetics, metabolism or imaging distribution of compounds. Analogous to the microdose trials described in the EMEA Position Paper, after the administration of very low doses of the novel molecular entities to few humans, very sensitive assay methods (e.g. PET, AMS) are needed to explore human pharmacokinetics and tissue distribution. The concept of microdose defined by the US FDA is analogous to the EMEA: one-hundredth of the proposed pharmacological dose from *in vivo* and/or *in vitro* data with a maximum dose of ⩽100 *μ*g (or ⩽30 nanomol for protein products). Considering that such low (sub-pharmacological) doses are not expected to have clinically significant toxic potential, reduced preclinical safety packages, bulk drug synthesis (CMC-chemistry, manufacturing and control requirements) and easier formulation are justified in some cases.

Analogous to the EMEA recommendations, extended single-dose toxicity studies in both genders of a single mammalian species to establish a dose inducing a minimal toxic effect or a safety margin are considered sufficient to support a human microdose study if justified by *in vitro* data. In contrast to the EMEA indications, routine genetic toxicology and safety pharmacology tests are not required.

As intended by the US FDA, microdose studies should be utilised in oncology, primarily for imaging end points. However, the Phase 0 guidance provides the opportunity to assess pharmacokinetic and pharmacodynamic parameters as well.

*Clinical trials to study pharmacologically relevant doses* are the second type of Phase 0 studies described in the US FDA's guidance. They are aimed to explore pharmacologic effects of a test molecule. These Phase 0 studies have no therapeutic or diagnostic purpose and are not meant to evaluate the maximally tolerated dose. The support of a preclinical safety package is more extensive than that of microdose studies, but still less than is required for traditional Phase I investigational new drug studies. Phase 0 studies to investigate pharmacologically active doses may include repeat dose clinical trials lasting up to 7 days.

Other Phase 0 trials described in the US FDA's guidance are *clinical studies of MOAs related to efficacy*. They are designed to evaluate the pharmacodynamic effect of a new drug that is expected to be correlated with its clinical activity. Typically, these Phase 0 trials present biomolecular end points, such as the rate of an enzyme inhibition or the degree of saturation of a receptor. These clinical studies of MOAs related to efficacy have been proposed as particularly useful in the oncology field for the evaluation of molecularly targeted anticancer agents. Indeed, according to the guidance, based on careful preclinical studies and appropriate assay development, a single dose of a targeted agent can be delivered with minimal risk (since such agents have been developed for chronic administration) at a level that has the potential to have a pharmacodynamic effect in tumour tissues in patients. This is expected to speed up drug development timelines by early evaluation of target effects and the usefulness of the assay systems for such targets.

To explore feasibility and utility of the Phase 0 strategy, after the publication of the US FDA guidelines, the National Cancer Institute (NCI, 2006) started the NCI Phase 0 programme and currently a Phase 0 pharmacokinetic and pharmacodynamic study of ABT-888, an inhibitor of poly(ADP-ribose) polymerase (PARP), in patients with refractory solid tumours and lymphoid malignancies, is ongoing (http://clinicaltrials.gov/ct/show/NCT00347633). This non-therapeutic study is designed to explore the pharmacokinetics, pharmacodynamics, side effects and safety of ABT-888. The programme will evaluate the real impact of the early-in-humans investigational new drug studies on drug development.

## POTENTIAL ADVANTAGES AND DISADVANTAGES OF PHASE 0 STUDIES

The Phase 0 trials were designed to improve the drug development pathway.

Phase 0 trials may shorten time periods to first-in-human studies through an earlier evaluation of human pharmacology of a test substance. The big advantage of Phase 0 trials is the possibility to explore pharmacokinetic and pharmacodynamic profiles of candidate drugs in humans (such as bioavailability, metabolism, tissue distribution), thus potentially helping in the early selection of promising compounds for further development before traditional Phase I trials. Indeed, one of the major limitations in anticancer drug development is the poor correlation between preclinical and clinical pharmacokinetics and pharmacodynamics. The early evaluation in humans of the candidate drug could potentially help in accelerating the selection of promising compounds, thus increasing the efficiency of the drug development process. The bioreductive agent EO9 was developed in Phase I; however, its pharmacokinetics in plasma showed extensive and rapid degradation to inactive metabolites, making the drug unsuitable for intravenous application (Schellens *et al*, 1994). The time-consuming and costly Phase I study could have been prevented if a Phase 0 study would have been performed. In addition, inactive treatment of patients who took part in that study could have been more limited.

Considering the reduced risk of human toxicity owing to the low dose of the test drug administered and the short time period of drug exposure in Phase 0 trials, the preclinical safety packages, animal use, amount of test drug, development time and costs will be reduced.

However, Phase 0 trials may get drugs faster into the clinic, but there are still concerns whether they can significantly shorten the drug development process, as they will not replace the traditional dose escalation, safety, tolerance and efficacy studies.

Other limitations relate to the lack of therapeutic intent of Phase 0 trials. The lack of therapeutic benefit is justified by the risk for the patient that is clearly less in a single-dose Phase 0 trial than in a standard dose-escalation Phase I study. However, in Phase I trials, although developed without therapeutic intent, there is on average a low response rate of about 5% (Von Hoff and Turner, 1991), whereas in Phase 0 trials no therapeutic gain can be expected. Therefore, besides the ethical issue, the motivation of patients to participate in a Phase 0 trial may significantly affect the success rate of these trials. Similar to several Phase I studies, in Phase 0 trials, serial tumour biopsies may need to be obtained or other invasive procedures may be necessary. Invasive procedures can be justified by the fact that more effort should be undertaken to develop decisive pharmacodynamic assays. However, it is difficult to envisage that an end-stage patient will consider to undergo multiple hospital admissions, multiple pharmacokinetic samplings and serial tumour biopsies without any potential benefit. If safe enough, the evaluation of the pharmacokinetics of a drug candidate can be conducted in healthy volunteers; however, pharmacodynamic measurements in tumour tissues and studies with cytotoxic agents have to be evaluated in patients.

Furthermore, participation in a Phase 0 trial might delay or exclude the patient from other clinical trials that may have some, although little, therapeutic intent. Although the total duration of a patient's participation in a Phase 0 trial is short and is expected to last no longer than 1 week, not only the preparation for a subsequent clinical study may be delayed but also a wash-out period may need to be taken into consideration. These issues may significantly impair patient recruitment, thus influencing the feasibility of Phase 0 trials.

There are other limitations regarding microdose studies. The US FDA guidance states that microdose studies should be utilised in oncology, primarily for imaging end points. However, as intended by the EMEA's Position Paper, microdose studies could be used to evaluate human plasma pharmacokinetics and/or distribution properties or the receptor selectivity profile of new drug candidates. In this case, an important question is whether there is a good correlation between the pharmacokinetics of a microdose and of a therapeutically relevant dose. In a study conducted by the Consortium for Resourcing and Evaluating AMS Microdosing, five drugs for which it was difficult to predict human pharmacokinetics were administered to subjects at a microdose and at a therapeutic dose level. Of the five drugs evaluated, microdose measurements predicted the pharmacokinetics of three compounds well when administered at therapeutic levels and gave important metabolism data for one drug. An approximately 70% correspondence between the pharmacokinetics of a microdose and of a pharmacologically relevant dose was found. Microdosing appears to be appropriate for compounds (e.g. small organic molecules, peptide and protein therapeutics) with linear pharmacokinetics, small molecules with short half-lives or compounds that are metabolised and that have a rapid dissociation of binding to their target. In contrast, microdosing was not predictive for molecules with nonlinear pharmacokinetics and/or with high-affinity binding to their targets (Lappin and Garner, 2003; Garner, 2005; Wilding and Bell, 2005; Singh, 2006; Lappin *et al*, 2006).

Another important concern is that for predicting anticancer activity, there are few validated biomarkers. Pharmacological, biological or imaging measurements at very low exposure levels are required, but unfortunately few reliable and validated assays are currently available even for most approved targeted cancer drugs. Moreover, along with novel therapeutic targets, more potent anticancer molecules have been discovered recently. This implies the use of lower doses than of traditional anticancer agents, thus potentially creating significant problems regarding the bioanalytical techniques. Indeed, for microdose studies, very sensitive analytical methods (such as PET or AMS) are needed, and currently these techniques are not routinely available (Gupta *et al*, 2002). This factor may further limit the application of Phase 0 microdose studies.

## CONCLUSIONS AND PERSPECTIVES

In its recent guideline, the US FDA gave clear prerequisites, principles and instructions for the execution of Phase 0 trials. Also the recently modified EMEA note for guidance on the development of anticancer medicinal products, supported by other EMEA guidelines, gives clear instructions about the prerequisites and execution of early clinical trials with anticancer agents. The EMEA currently prepares a guideline for Phase 0 trials that will supplement the existing guidelines.

Phase 0 trials are expected to improve the efficiency of (anticancer) drug development and to reduce costs. The rate of failure of drugs entering Phase I clinical trials should come down as a proof that Phase 0 trials are beneficial. Furthermore, Phase 0 trials should contribute to fast-track and safe drug development. Future experience should tell whether these expectations would be fulfilled. Therefore, critical evaluation of Phase 0 trials is necessary, especially in terms of safety. One should prevent that Phase 0 trials will be used to circumvent proper safety assessment in the preclinic as well as proper assessment of proof of concept of antitumour activity of a clinical candidate drug before first-in-man studies are being performed. Furthermore, it should be critically monitored whether Phase 0 trials contribute to faster drug development without putting patients at unacceptable risks.

Execution of Phase 0 trials may be hampered by ethical reasons as well as by the willingness of patients to take part in these trials that will have no therapeutic benefit to them.

Despite the opportunities provided by Phase 0 trials, new animal models with humanised biotransformation systems and drug transporters, and animals bearing genetically modified tumours better reflecting human tumour biology and drug disposition (the so-called ‘mouse clinic’), could significantly improve the drug development process. By using these models, it is expected that more efficient, faster and less costly drug development is achieved, especially by better preclinical selection of clinical candidates based on more stringent assessment of proof of concept as well as by selection of clinical candidates with better pharmacological profiles and by better definition of the target population of patients during clinical development.
